# Au@CdS Core–Shell Nanoparticles‐Modified ZnO Nanowires Photoanode for Efficient Photoelectrochemical Water Splitting

**DOI:** 10.1002/advs.201500135

**Published:** 2015-07-17

**Authors:** Chun Xian Guo, Jiale Xie, Hongbin Yang, Chang Ming Li

**Affiliations:** ^1^ Institute for Clean Energy and Advanced MaterialsSouthwest UniversityChongqing00715P.R.China; ^2^ Chongqing Key Laboratory for Advanced, Materials and Technologies of Clean Electrical Power SourcesChongqing400715P.R. China; ^3^ School of Chemical and Biomedical EngineeringNanyang Technological UniversitySingapore637457Singapore

**Keywords:** CdS, core–shell, nanowire, photoanode, photoelectrochemical water splitting

## Abstract

Hydrogen production from water splitting using solar energy based on photoelectrochemical (PEC) cells has attracted increasing attention because it leaves less of a carbon footprint and has economic superiority of solar and hydrogen energy. Oxide semiconductors such as ZnO possessing high stability against photocorrosion in hole scavenger systems have been widely used to build photoanodes of PEC cells but under visible light their conversion efficiencies with respect to incident‐photon‐to‐current conversion efficiency (IPCE) measured without external bias are still not satisfied. An innovative way is presented here to significantly improve the conversion efficiency of PEC cells by constructing a core–shell structure‐based photoanode comprising Au@CdS core–shell nanoparticles on ZnO nanowires (Au@CdS‐ZnO). The Au core offers strong electronic interactions with both CdS and ZnO resulting in a unique nanojunction to facilitate charge transfer. The Au@CdS‐ZnO PEC cell under 400 nm light irradiation without any applied bias provides an IPCE of 14.8%. Under AM1.5 light illumination with a bias of 0.4 V, the Au@CdS‐ZnO PEC cell produces H2 at a constant rate of 11.5 μmol h^−1^ as long as 10 h. This work provides a fundamental insight to improve the conversion efficiency for visible light in water splitting.

## 1 Introduction

This is an open access article under the terms of the Creative Commons Attribution License, which permits use, distribution and reproduction in any medium, provided the original work is properly cited.

Since using a TiO_2_ photoanode to split water by Fujishima and Honda, researchers have been attempting to develop efficient photo­anodes for photoelectrochemical (PEC) water splitting and thus producing hydrogen.^1^, ^2^, ^3^, ^4^, ^5^, ^6^ To achieve high energy conversion efficiency, photoanodes should effectively harvest the sunlight and facilitate charge transfer while possessing long‐term stability.^7^, ^8^, ^9^ ZnO and TiO_2_, oxide semiconductors possess high stability against photocorrosion in hole scavenger systems, and have been widely used as photoanodes.^10^, ^11^ Nevertheless, they both have a wide band gap (e.g., ≈3.4 eV for ZnO at room temperature) and mainly utilize the UV light that is only around 5% of the sunlight spectrum. To effectively harvest the sunlight, in particular, to adsorb more visible light, narrow band gap species/semiconductors have been employed to sensitize the ZnO and TiO_2_ photoanodes. Quantum dot‐sensitized ZnO and carbon‐doped TiO_2_ photo­anodes have been investigated to improve PEC water splitting efficiency.^12^, ^13^ However, for currently developed ZnO and TiO_2_ photoanodes under visible light (*λ* ≥ 400 nm) the PEC efficiency with respective to incident‐photon‐to‐current conversion efficiency (IPCE) measured without any applied external bias is still lower than 10.0%.^14^, ^15^, ^16^, ^17^, ^18^, ^19^, ^20^ One reason for the low conversion efficiency is the limited charge transfer rate resulting in relatively high charge recombination.^21^, ^22^ The development of photoanodes with favorable structure/junction to direct charge transfer for high‐efficiency PEC water splitting cells is essential.

In this work, we develop a core–shell structure to facilitate charge transfer of ZnO‐based photoanode, which comprises core–shell Au@CdS nanoparticles anchored on ZnO nanowires (Au@CdS‐ZnO) that were grown on F‐doped SnO_2_ glass. The core–shell structure of Au@CdS is found to form a desired nanojunction between CdS and ZnO to greatly promote charge transfer, thus significantly enhancing the IPCE efficiency of the PEC cells to 14.8% measured with a two‐electrode configuration and without any applied bias. The efficiency is much higher than that of 9.5% for PEC cells in the absence of the core–shell structure, and is the best among all ZnO‐based two‐electrode PEC cells.

## 2 Results and Discussion

### 2.1 Photoanode Fabrication and Characterizations

**Figure 1:**
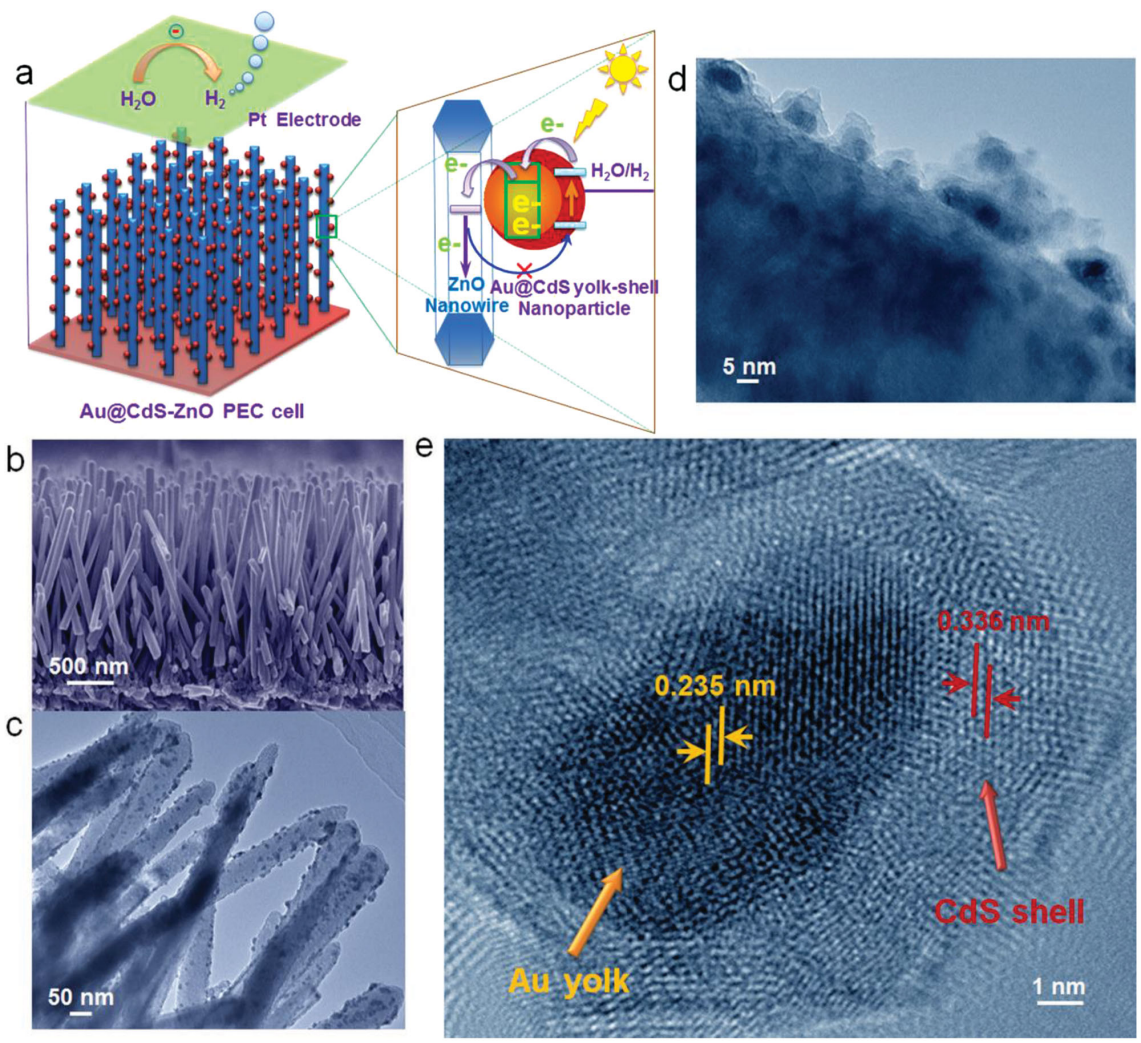
a) Au@CdS‐ZnO PEC cell configuration and its photoinduced charge transport/transfer process. b) Cross‐section SEM image of ZnO nanowire arrays directly grown on F‐doped SnO_2_ glass. c,d) TEM images with different magnifications of Au@CdS‐ZnO. e) HRTEM image showing the detailed structure of Au@CdS core–shell on ZnO.

**Figure** **1**a shows the configuration of the core–shell structure‐based photoanode and its PEC cell with a Pt counter electrode. The photoanode is composed of core–shell Au@CdS nanoparticles anchored on ZnO nanowires (Au@CdS‐ZnO) that were grown on F‐doped SnO_2_ glass, a transparent and conducting substrate. The ZnO nanowires have an average length of 2 μm and a diameter around 100 nm, and possess a surface density as high as ≈2 × 10^9^ wire cm^−2^ (Figure 1b and Figure S1, Supporting Information). To fabricate the core–shell Au@CdS nanoparticles‐modified ZnO nanowires photoanode, Au nanoparticles were grown on ZnO by chemical reduction of chloroauric acid with sodium citrate, followed by depositing CdS shell on Au nanoparticles via a sequential chemical bath deposition. The Au@CdS nanoparticles with an average size of 10 nm are uniformly distributed on the surface of ZnO nanowires (Figure 1c,d) while having a well‐defined core–shell structure, in which the Au core locates between the ZnO and CdS (Figure 1e and Figures S2 and S3, Supporting Information). The Au core has an average size of around 5 nm and a lattice spacing of 0.235 nm, indexed to the (111) reflections of face‐centered cubic Au.^23^ The CdS shell is quite thin and has a lattice spacing of 0.336 nm, corresponding to the (220) plane of the cubic phase of CdS.^24^

The thickness of the CdS shell can be controlled by the number of the sequential chemical bath deposition cycles (Figure S4, Supporting Information). It has a growth rate of around 0.3 nm cycle^−1^ when the cycle number is no more than 15 cycles. Specifically, at the eighth cycle the CdS shell has an average thickness of 2.5 nm. However, when the cycle number further increases (>15), CdS clusters begin to appear. The composition of the Au@CdS‐ZnO was examined by energy dispersive X‐ray spectroscopy (EDS) (Figure S5, Supporting Information), which further confirms that the photoanodes comprise ZnO, Au, and CdS. In addition, the molar ratio of Cd/S was determined to be around 1. In respect to the formation process of CdS shell on Au core, it is very likely that S^2−^ ions from their salt solution are selectively adsorbed on Au nanoparticle surface followed by binding of the adsorbed S^2−^ ions on Au with Cd^2+^ ions to form CdS shell. These characterization results demonstrate the successful fabrication of the Au@CdS‐ZnO photoanode.

### 2.2 PEC Water Splitting Performance Investigation

**Figure 2:**
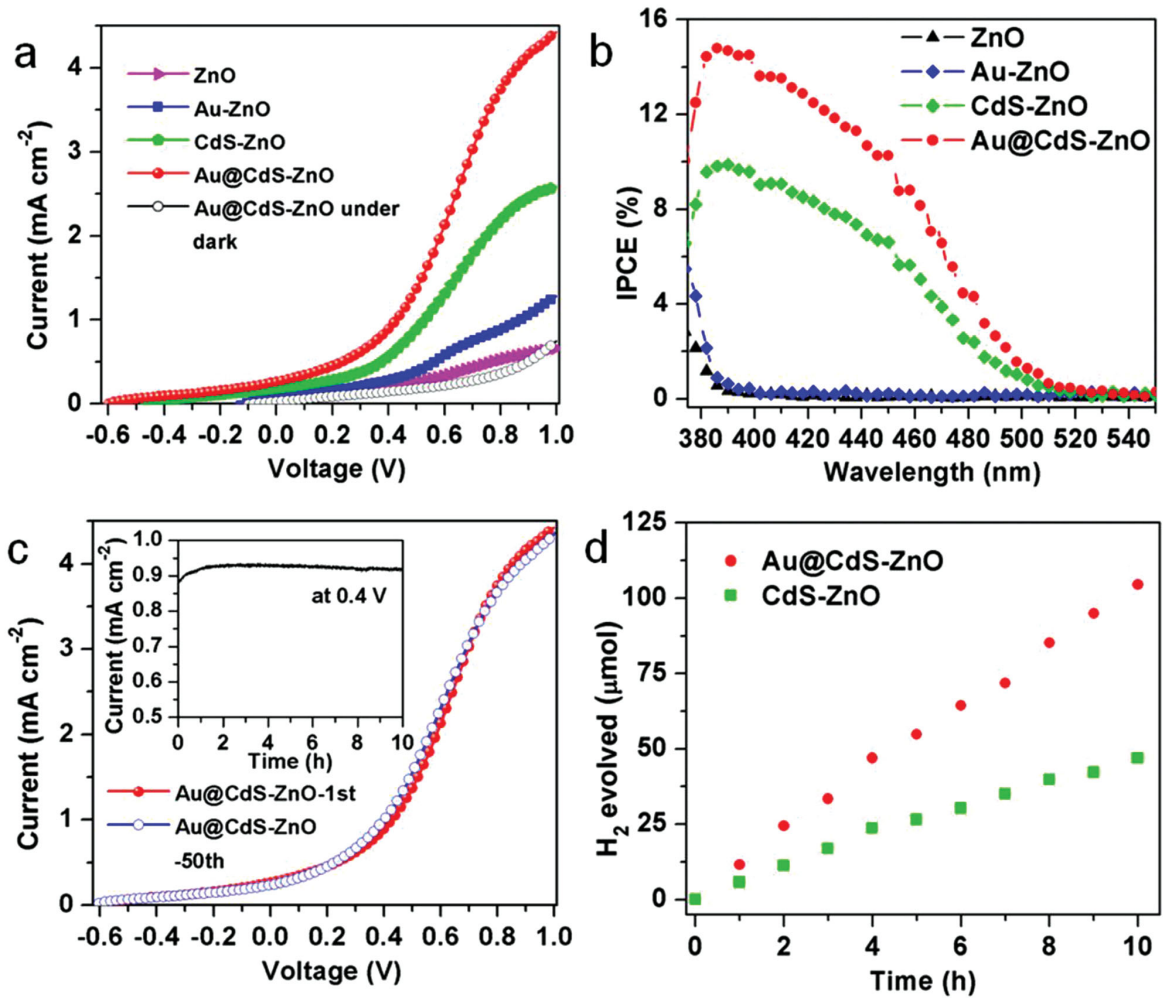
a) Linear‐sweep voltammograms (LSVs) of PEC cells made up of two‐electrode system comprising a photoanode and a Pt counter electrode. b) Incident‐photon‐to‐current conversion efficiency (IPCE) spectra of the PEC cells. No external bias is applied. c) Stability of the Au@CdS‐ZnO PEC cell. Inset is the plot of photocurrent density versus time at a bias of 0.4 V. d) Time courses of H_2_ evolution of CdS‐ZnO and Au@CdS‐ZnO PEC cells at a bias of 0.4 V under AM1.5 light irradiation. Aqueous electrolyte composing of 0.25 m Na_2_S and 0.35 m Na_2_SO_3_ was used for all PEC cells with two‐electrode configuration.

PEC water splitting performance was evaluated using a two‐electrode PEC cell (Figure 1a) with an aqueous electrolyte composing of 0.25 m Na_2_S and 0.35 m Na_2_SO_3_, where Na_2_S/Na_2_SO_3_ in the solution functions as a hole scavenger. Under operation, the photoanode was illuminated under light of 100 mW cm^−2^. As shown in **Figure** **2**a, the plain ZnO PEC cell provides an open‐circuit voltage (*V*_oc_) of 0.06 V and a short‐circuit photocurrent (*I*_sc_) of 0.035 mA cm^−2^, showing relatively low PEC activity, which should be due to its poor absorption of visible light. CdS‐ZnO PEC cell exhibits an enhancement in PEC activity with a *V*_oc_ of 0.45 V and an *I*_sc_ of 0.174 mA cm^−2^, which is respective eight and five times of that of plain ZnO photoanode‐based PEC cell. The enhancement should come from the improved visible light absorption of CdS. More importantly, Au@CdS‐ZnO PEC cell further increases the *I*_sc_ to 0.273 mA cm^−2^, a 60% increase than the CdS‐ZnO PEC cell. In addition, Au@CdS‐ZnO PEC cell exhibits a *V*_oc_ of 0.60 V, 0.15 V higher than that of CdS‐ZnO PEC cell but a dark scan for the Au@CdS‐ZnO PEC cell shows a negligible *V*_oc_ and *I*_sc_. These results indicate that the use of Au greatly improves the PEC activity. On the other hand, a control device of Au‐ZnO PEC cell displays a *V*_oc_ of 0.12 V and an *I*_sc_ of 0.063 mA cm^−2^, close to those of ZnO PEC cell, suggesting that the significantly improved PEC activity of the Au@CdS‐ZnO PEC cell is mainly attributed to the favorable interaction between Au and CdS of the Au@CdS core–shell structure. The CdS shell thickness has an effect on the activity of the Au@CdS‐ZnO PEC cell. The photocurrent density increases with the CdS shell thickness, and reaches the maximum at the thickness of around 2.5 nm while decreasing when the shell thickness further increases (Figure S6, Supporting Information).

Performance of the PEC cells was further investigated by recording IPCE spectra, which were measured under monochromatic illumination as a function of incident photon wavelength using the two‐electrode configuration without any applied external bias. The IPCE was calculated by the equation: IPCE = (1240*I*)/(*λJ*_light_),^25^ where *I* is the photocurrent density, *λ* the incident light wavelength, and *J*_light_ the measured irradiance. Compared with that of the ZnO PEC cell, IPCE of the Au‐ZnO PEC cell was enhanced at light with wavelength less than 400 nm while no obvious enhancement in the light region with wavelength larger than 400 nm (Figure 2b). At a wavelength of 400 nm, Au@CdS‐ZnO PEC cell has an IPCE as high as 14.8%, which is much higher than that of the Au‐ZnO (0.3%), CdS‐ZnO (9.5%) PEC cells, and also Au‐CdS‐ZnO PEC cell (11.2%) (Figure 2b and Figure S8, Supporting Information). It is noted that the IPCE value of 14.8% is higher than the sum (9.8%) of IPCE value of the Au‐ZnO and CdS‐ZnO PEC cells, and among the highest ones for all ZnO‐based PEC cells.^14^, ^15^, ^16^, ^17^, ^18^, ^19^, ^20^ We have also measured PEC performance of the Au@CdS‐ZnO photoanode using a three‐electrode configuration with an Ag/AgCl reference electrode and a Pt counter electrode (Figure S9, Supporting Information), finding out that the photoanode exhibits a *V*_oc_ of 1.0 V and *I*_sc_ of more than 2.5 mA cm^−2^, which are much higher than those (*V*_oc_ of 0.6 V and *I*_sc_ of 0.273 mA cm^−2^) of the same photoanode based on the two‐electrode configuration. Nevertheless, to be practically used, the two‐electrode system is still utilized in the following experiments. In a long term operation, the stability of a PEC cell is also critical. The PEC activity of the Au@CdS‐ZnO PEC cell is almost identical after 50 cycles (Figure 2c). In addition, under illumination at a bias of 0.4 V, the cell retains 98.5% of its original response after 10 h operation, demonstrating good stability. Under a bias of 0.4 V, the amount of H_2_ generated of the Au@CdS‐ZnO PEC cell under light illumination was detected using on‐line gas chromatograph, producing about 11.5 μmol h^−1^ H_2_, which is higher than that of CdS‐ZnO PEC cell (5.0 μmol h^−1^ H_2_) (Figure 2d) and Au‐CdS‐ZnO (8.3 μmol h^−1^ H_2_) (Figure S10, Supporting Information). The Au@CdS‐ZnO photoanode well retained its structure after the H_2_ evolution testing (Figure S11, Supporting Information), further evidencing the good stability.

**Figure 3:**
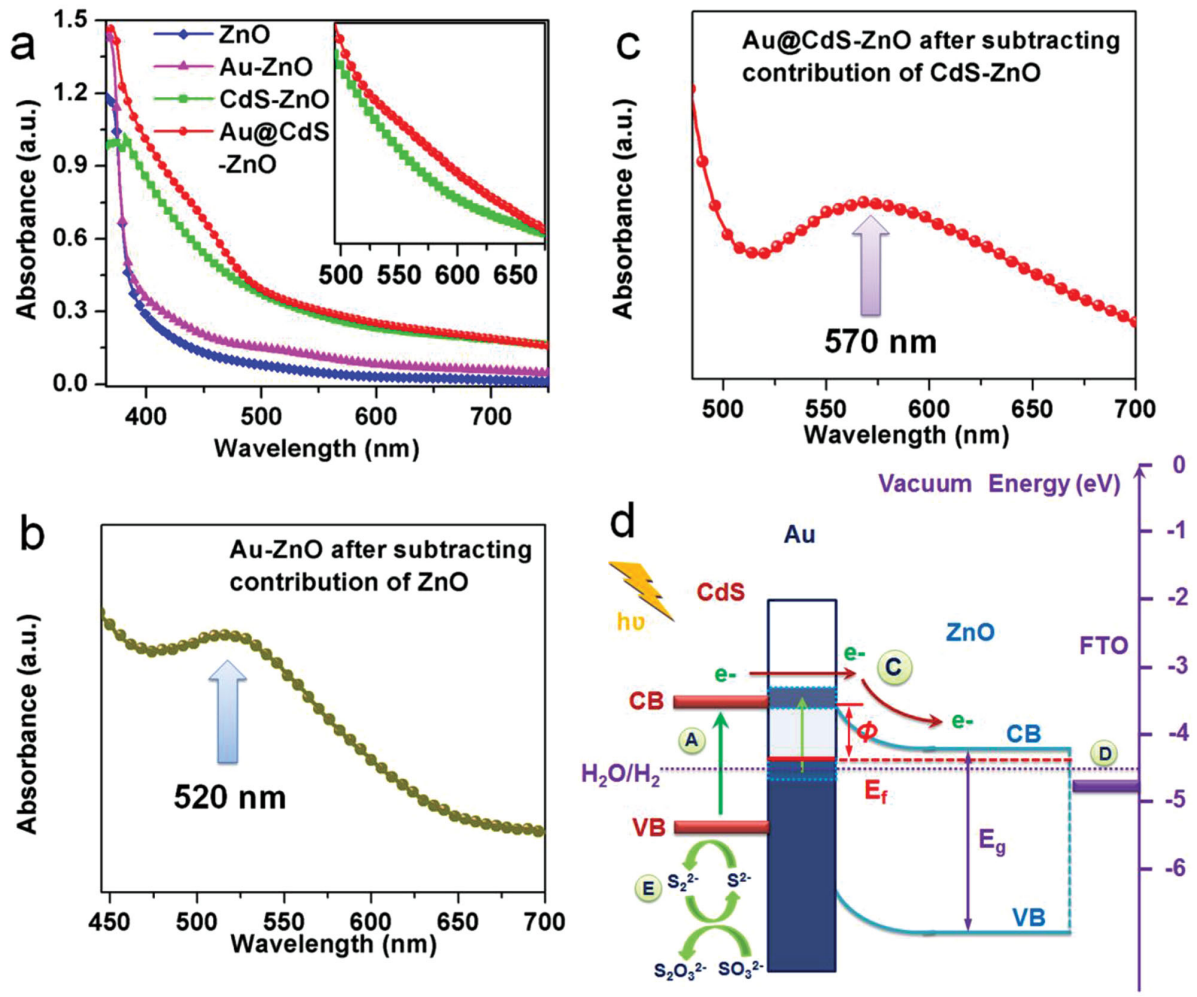
a) Optical absorption spectra of photoelectrodes. The inset is enlarged spectra of CdS‐ZnO and Au@CdS‐ZnO. b) Optical absorption spectrum of Au‐ZnO photoelectrode after subtracting contribution of ZnO. c) Optical absorption spectrum of Au@CdS‐ZnO photoelectrode after subtracting contribution of CdS‐ZnO. d) Electron transfer path of Au@CdS‐ZnO. Process A: photon absorption and electron excitation of CdS shell; B: plasmon resonance of Au core; C: energetic electron transfer from CB of CdS shell over the potential energy barrier of Au into ZnO; D: electron conduction as a majority carrier within the ZnO to the FTO glass; E: reduction of the photogenerated holes. Φ for the Schottky barrier, *E*_f_ for the Fermi energy, *E*_g_ for the ZnO bandgap, and CB and VB for conduction band and valence band, respectively.

To explore the electronic interaction between Au and CdS, light adsorption for various photoelectrodes was investigated. As illustrated in **Figure** **3**a, ZnO photoelectrode only shows light adsorption in the UV region resulted from its wide band gap. Different from ZnO photoelectrode, Au‐ZnO photoelectrode exhibits a weak band in the visible region between 500 and 600 nm. By subtracting contribution of ZnO from the optical absorption spectrum of Au‐ZnO photoelectrode, the absorption band centered at 520 nm can be clearly seen (Figure 3b), which is consistent with the observed absorption band of the plain Au nanoparticles (Figure S12, Supporting Information) and should be attributed to Plasmon resonance of the Au NPs,^16^ suggesting a strong electronic interaction between the Au and ZnO. The use of CdS with a relative narrow bandgap improves visible light adsorption of both CdS‐ZnO and Au@CdS‐ZnO photoelectrodes. By subtracting contributions of CdS and ZnO from the optical absorption spectrum of Au@CdS‐ZnO, a clear but broad band centered at 570 nm was observed (Figure 3c). There is a red‐shift of 50 nm for Au in the Au@CdS‐ZnO in comparison with Au in the Au‐ZnO. The red‐shift should be resulted from the strong electronic interaction between Au and CdS.^26^ The good interface between Au and CdS can be directly seen from the high‐resolution transmission electron microscopy (HRTEM) image (Figure S7, Supporting Information). The strong electronic interaction between Au and both ZnO and CdS should play an important role in performance enhancement of the Au@CdS‐ZnO PEC cell.

### 2.3 PEC Performance Enhancement Discussion

Fundamentally, for photoanode‐based PEC cells, the conversion efficiency depends on light absorption and photoinduced charge separation/transfer process at a photoanode.^27^, ^28^, ^29^, ^30^, ^31^, ^32^ In the Au@CdS‐ZnO photoanode, Au has strong electronic interactions with both ZnO and CdS, thus promoting the formation of a well‐defined nanojunction with Au centered between ZnO and CdS (Figure 3d). With regard to the bandgap structure, Au has an electron work function of around 5.1 eV versus vacuum and ZnO has a bandgap of around 3.4 eV with conducting band and valence band at 4.2 and 7.6 eV versus vacuum, respectively. Thus, a Schottky barrier, *Φ*, approximately 0.9 V is formed at the Au and ZnO interface.^33^ Based on the Brus equation, CdS shell with a thickness of around 2.5 nm has a band gap of around 2.7 eV and a conducting band around 3.8 eV versus vacuum,^34^ ensuring the photoexcited donor level energetically above the Schottky barrier. Therefore, the Au@CdS‐ZnO photoanode with desired nanojunction is energetically favorable for charge transfer. The greatly improved charge transfer by the Au@CdS‐ZnO structure is also supported by its much lower charge transfer resistance than that of ZnO and CdS‐ZnO as in the electrochemical impedance spectroscopy measured at open circuit voltage under AM1.5 light irradiation (Figure S13, Supporting Information). During operation of the Au@CdS‐ZnO PEC cells under light illumination, it starts from light absorption by the CdS shell and plasmon resonance of Au core, giving rise to energetic electrons, most of which should come from CdS. These energetic electrons have energy above the Au Fermi energy, and are greater than the Schottky barrier height, thus favourably travelling through Au to direct charge transfer between CdS and ZnO and resulting in greatly improved conversion efficiency of Au@CdS‐ZnO PEC cells.

## 3 Conclusion

In summary, we report the development of an efficient Au@CdS core–shell nanoparticles‐ modified ZnO nanowires photoanode for PEC water splitting. Au core in the photoanode offers strong electronic interactions with both CdS and ZnO, leading to a favorable nanojunction to facilitate charge transfer of photoexcited electrons to ZnO. Under light illumination with a wavelength of 400 nm, the Au@CdS‐ZnO PEC cell with a two‐electrode configuration and without applied bias provides an IPCE as high as 14.8%, which is much higher than that of the Au‐ZnO (0.3%) and CdS‐ZnO (9.5%) PEC cells, and ranks the best among all ZnO‐based PEC cells that were measured under similar conditions. The Au@CdS‐ZnO PEC photoanode also has good stability with almost retained PEC activity after 50 cycle running under light illumination. Under AM1.5 light illumination with a bias of 0.4 V, the Au@CdS‐ZnO PEC cell can produce H_2_ with a constant rate of 11.5 μmol h^−1^ for 10 h. This work provides a facile way while offering fundamental insights to a direct charge transfer mechanism for highly efficient PEC cells.

## 4 Experimental Section

*Fabrications of Photoanodes*: FTO glasses (7 Ω per square) were first cleaned using acetone, ethanol, and deionized water, respectively. They were coated with 0.25 m zinc acetate by spin coating, followed by annealing at 350 °C for 30 min to form a layer of ZnO seeds. ZnO nanowires were grown by immersing ZnO seed‐coated fluorine doped tin oxide (FTO) glasses in solution containing 0.025 m zinc nitrate hydrate and 0.025 m hexamethylenetetramine for 6 h at 95 °C. The substrates were then rinsed with deionized water, and treated at 400 °C for 30 min. After cooling to room temperature, they were put into an oxygen plasma cleaner for 10 min and then immersed in a chloroauric acid (1 × 10^−3^ m) solution with a pH value of 9.0 adjusted by sodium citrate to grow Au nanoparticles. The resulting substrates were then repeatedly coated with CdS by sequential chemical bath deposition by immersing in four different beakers with 0.05 m CdCl_2_ solution, water, 0.05 m Na_2_S solution, and water, subsequently for 30 s each, resulting in Au@CdS‐ZnO. As a control material, Au‐CdS‐ZnO was fabricated by depositing CdS, followed by growing Au nanoparticles.

*PEC Water Splitting Experiments*: The PEC device was constructed with a two‐electrode configuration: a photoanode having an area of 1.2 cm^2^ and a platinum foam of 9.0 cm^2^ as the counter electrode. The cell with dimensions of 4 × 2 × 2 cm (*L* ×*W* ×*W*) was made by quartz glass. The electrolyte was 0.25 m Na_2_S and 0.35 m Na_2_SO_3_ aqueous solution. The experiments were conducted using a 150 W Xe lamp (filtered, *λ* > 350 nm) and the illumination intensity near the photoelectrode surface is 100 mW cm^−2^. Linear‐sweep voltammograms were recorded with an electrochemical analyzer (CHI Instruments). Electrochemical impedance spectroscopy spectra were recorded at open circuit voltage under AM1.5 light irradiation. Incident photon to current conversion efficiency measurement was performed under illumination with respect to a calibrated silicon diode. The monochromic light was supplied by xenon light illuminating through a Cornerstone monochromator. A chopper was placed after the monochromator and the signal was collected by Merlin lock‐in radiometry after amplification by the current preamplifier. Evolved H_2_ was detected by an online gas chromatograph (Tianmei GC7900 with a thermal conductivity detector; carrier gas: high‐purity nitrogen). All measurements were carried out at room temperature.

*Material Characterizations*: The morphology and structure of the materials were investigated by field emission scanning microscopy (SEM, JSM‐6700F) and transmission electron microscopy (TEM, JEM‐2010F, Japan). Compositions of the photoanode were determined by EDS (JSM‐6700F). The crystal structure was characterized by powder X‐ray diffraction (XRD, Bruker AXS X‐ray Diffractometer).

## Supporting information

As a service to our authors and readers, this journal provides supporting information supplied by the authors. Such materials are peer reviewed and may be re‐organized for online delivery, but are not copy‐edited or typeset. Technical support issues arising from supporting information (other than missing files) should be addressed to the authors.

Supplementary
