# Supplementary material for: Au@CdS Core–Shell Nanoparticles‐Modified ZnO Nanowires Photoanode for Efficient Photoelectrochemical Water Splitting
Source: Adv Sci (Weinh). 2015 Jul 17;2(12):1500135. doi: 10.1002/advs.201500135 (PMC5115296; doi:10.1002/advs.201500135)
Supplement: Supplementary file 1 — Supplementary [file ADVS-2-0q-s001.pdf]

Copyright WILEY-VCH Verlag GmbH & Co. KGaA, 69469 Weinheim, Germany, 2015.

Supporting Information

for *Adv. Sci.*, DOI: 10.1002/advs. 201500135

Au@CdS Core–Shell Nanoparticles-Modified ZnO Nanowires Photoanode for Efficient Photoelectrochemical Water Splitting

*Chun Xian Guo, Jiale Xie, Hongbin Yang, and Chang Ming Li**

Copyright WILEY-VCH Verlag GmbH & Co. KGaA, 69469 Weinheim, Germany, 2013.

Supporting Information

Au@CdS Core-Shell Nanoparticles-modified ZnO Nanowires Photoanode for Efficient Photoelectrochemical Water Splitting

*Chun Xian Guo, Jiale Xie, Hongbin Yang, and Chang Ming Li **

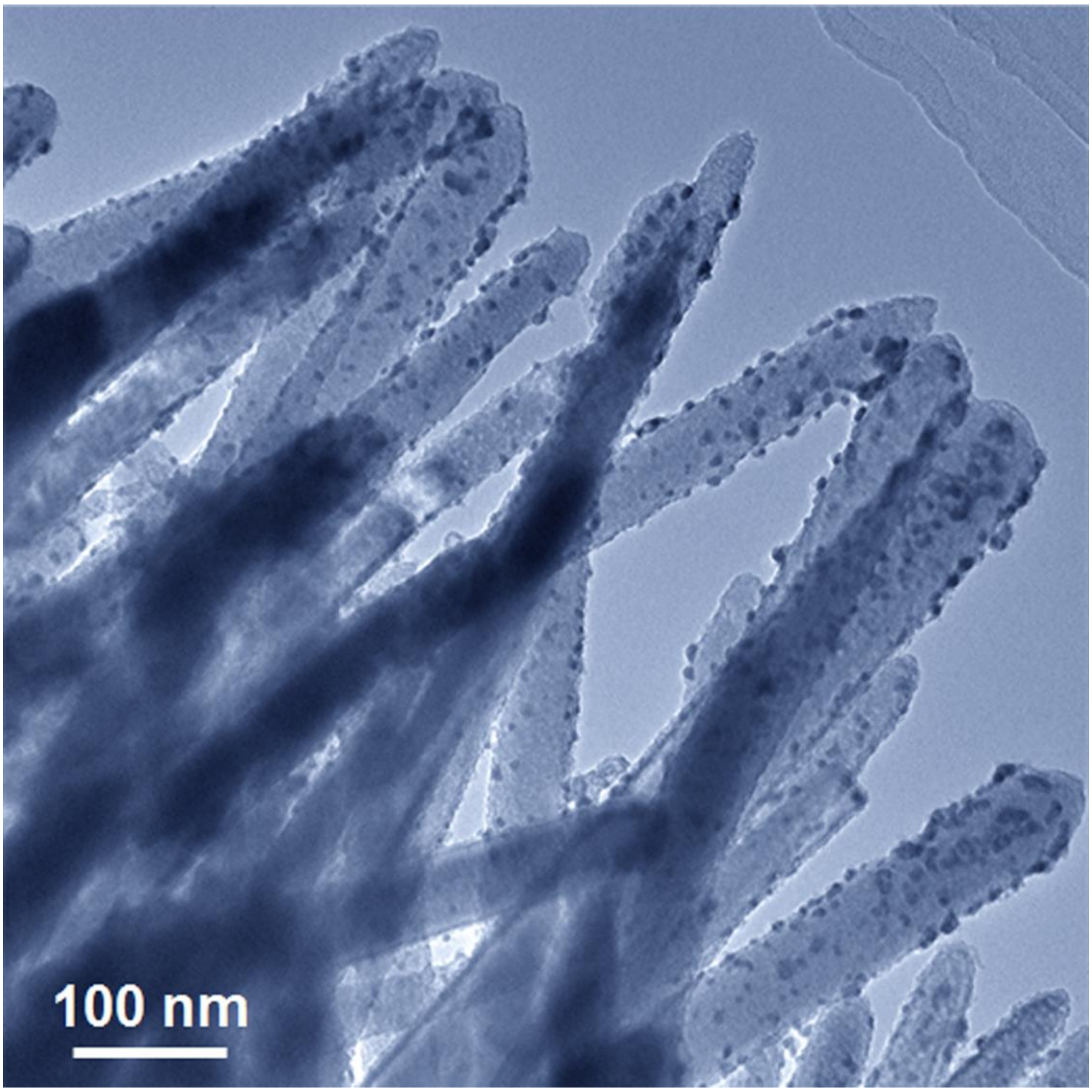

Figure S1. TEM image of Au@CdS-ZnO.

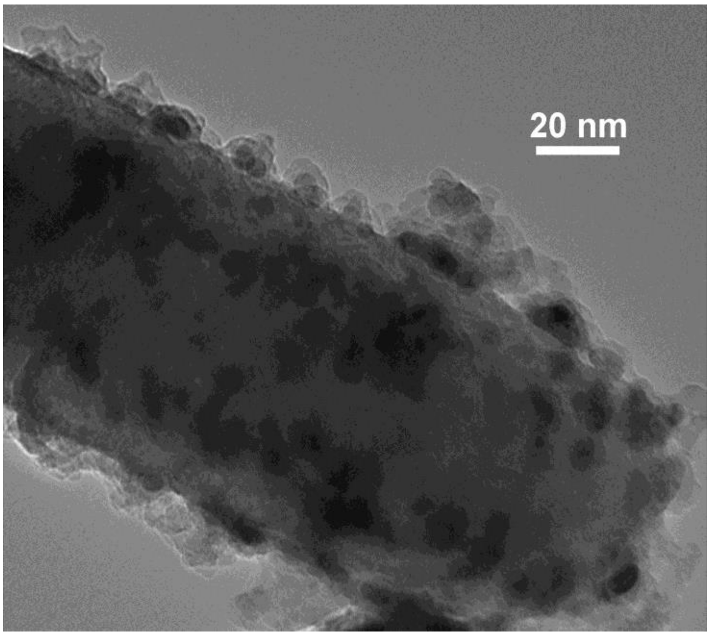

Figure S2. TEM image showing the Au@CdS core-shell nanoparticles anchored ZnO nanowire.

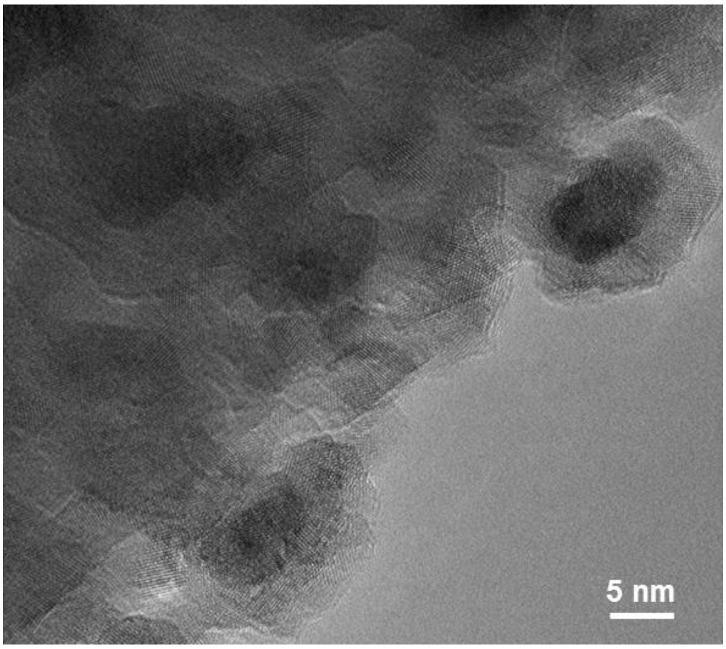

Figure S3. HRTEM image showing the detailed Au@CdS core-shell structure on ZnO.

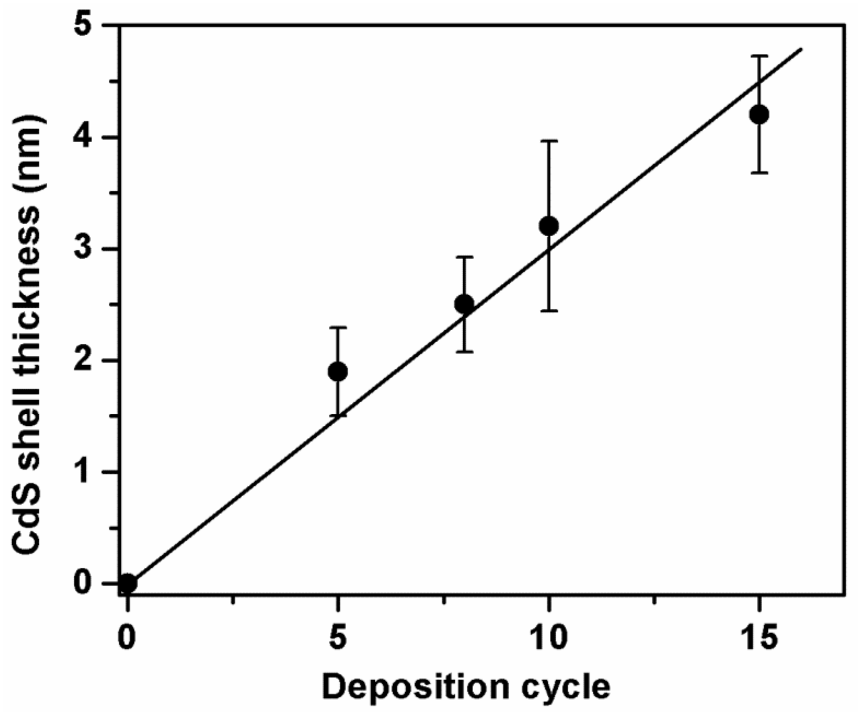

Figure S4. Relation between shell thickness of CdS and the sequential chemical bath deposition cycle.

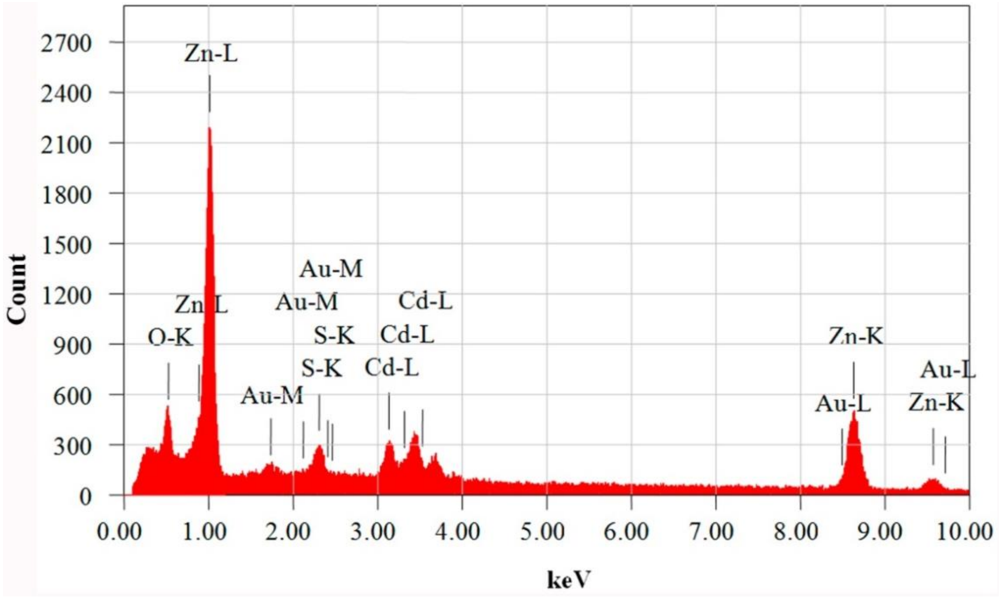

Figure S5. Energy dispersive X-ray spectroscopy spectrum showing the composition of the Au@CdS-ZnO.

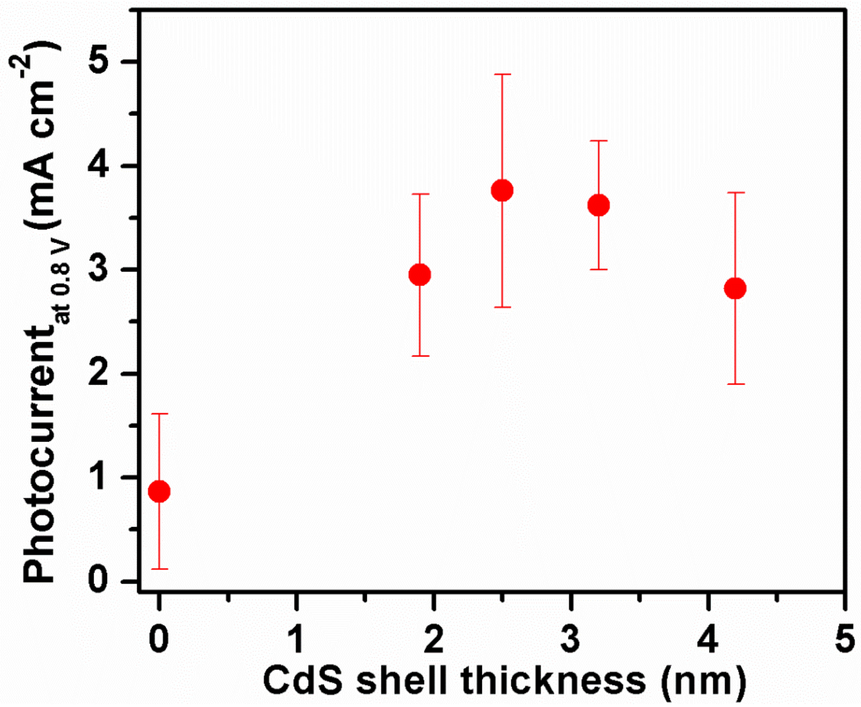

Figure S6. Dependence of photocurrent density at an applied bias of +0.8 V with CdS shell thickness. The error bars represent standard deviation.

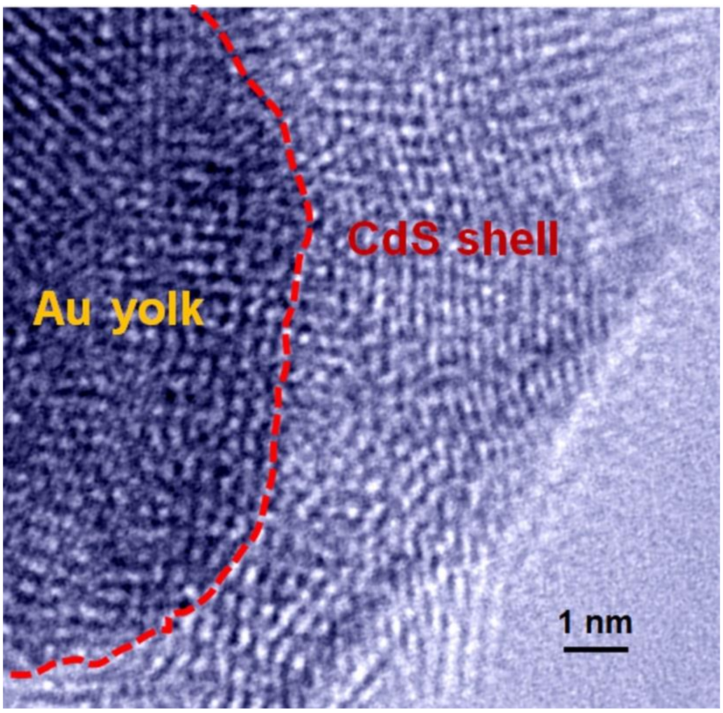

Figure S7. TEM image showing interface between Au and CdS of Au@CdS-ZnO.

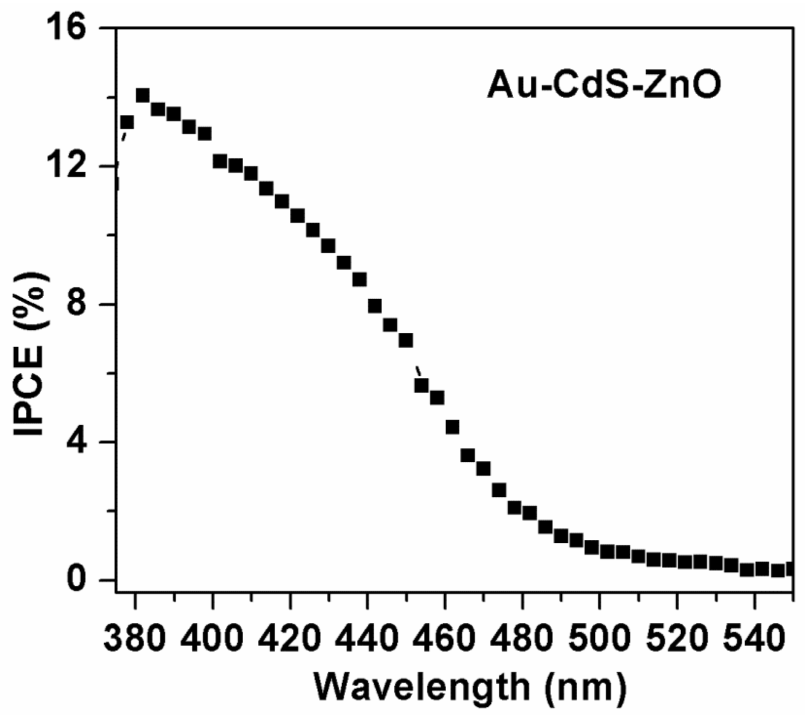

Figure S8. IPCE spectrum of the PEC cell of Au-CdS-ZnO. No external bias is applied.

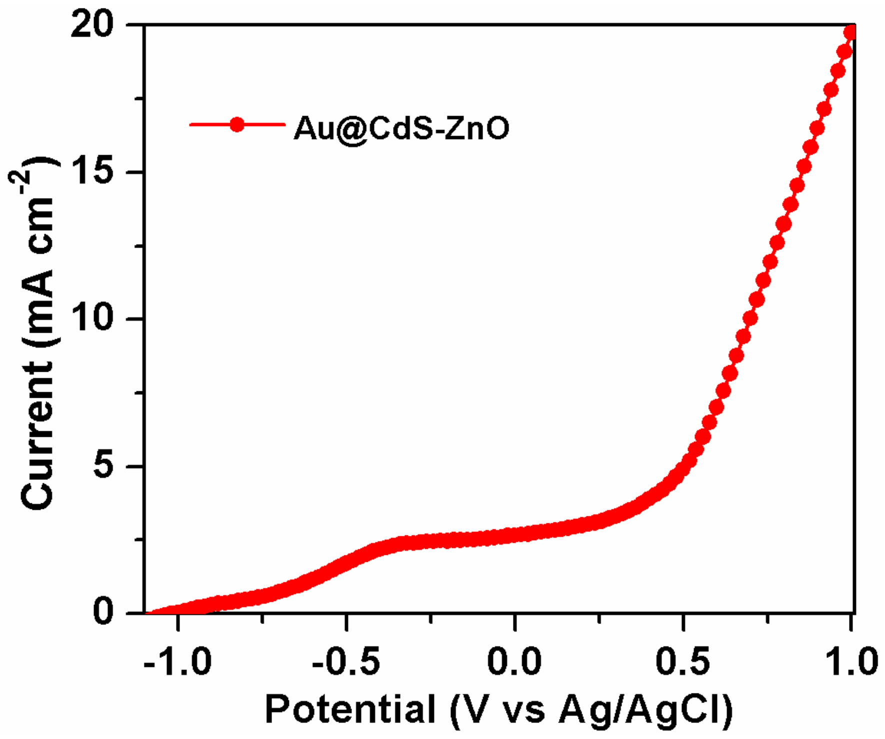

Figure S9. Linear-sweep voltammogram of PEC cell comprising the Au@CdS-ZnO photoanode, Ag/AgCl reference electrode and Pt counter electrode under AM1.5 light irradiation. Aqueous electrolyte composing of 0.25 M Na_2S and 0.35 M Na_2SO_3.

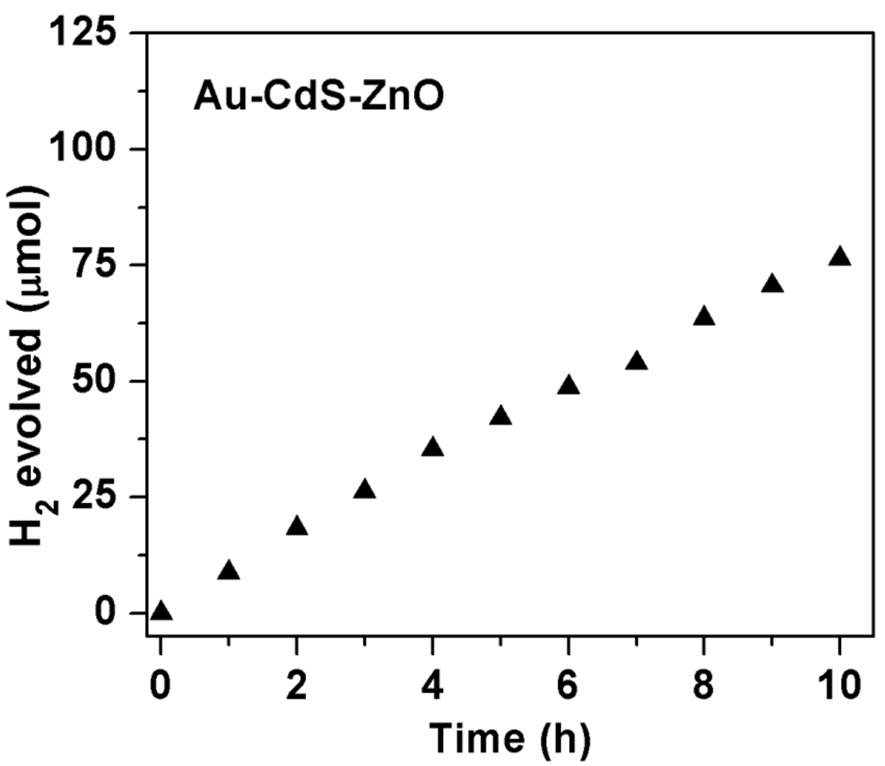

Figure S10. Time courses of H_2 evolution of Au-CdS-ZnO PEC cell at a bias of 0.4 V under AM1.5 light irradiation.

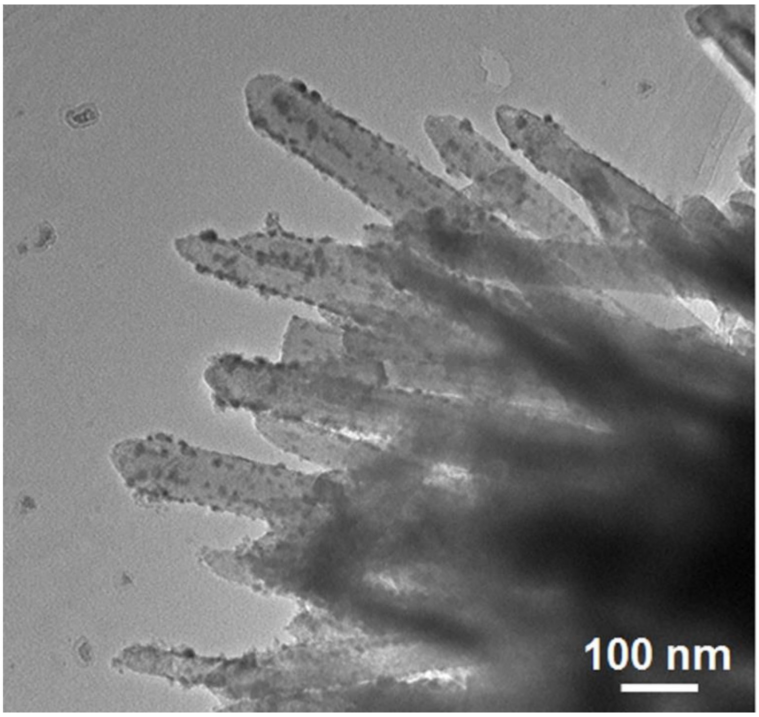

Figure S11. TEM image of Au@CdS-ZnO photoanode after H_2 evolution testing.

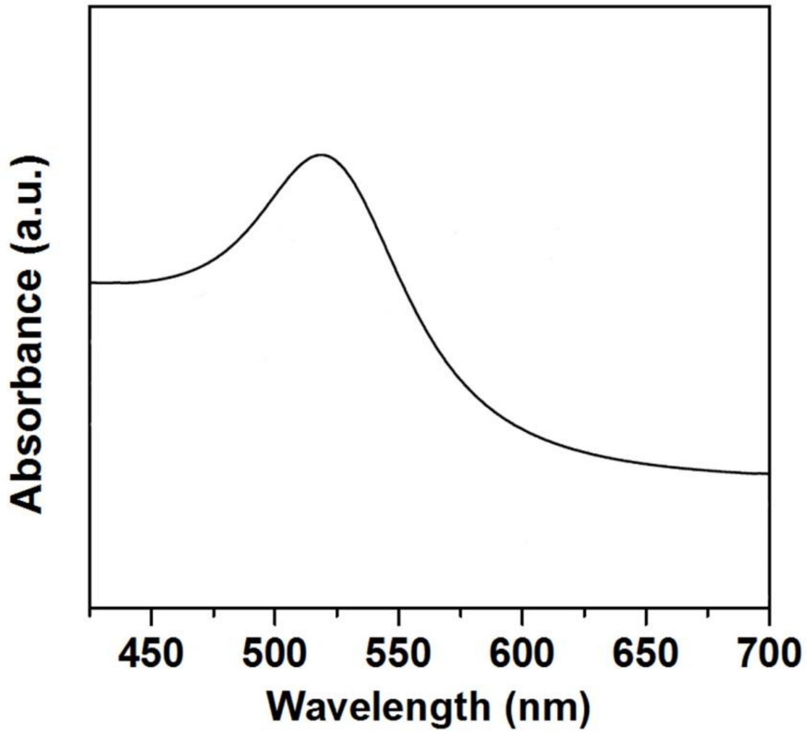

Figure S12. Optical absorption spectrum of aqueous solution of Au nanoparticles.

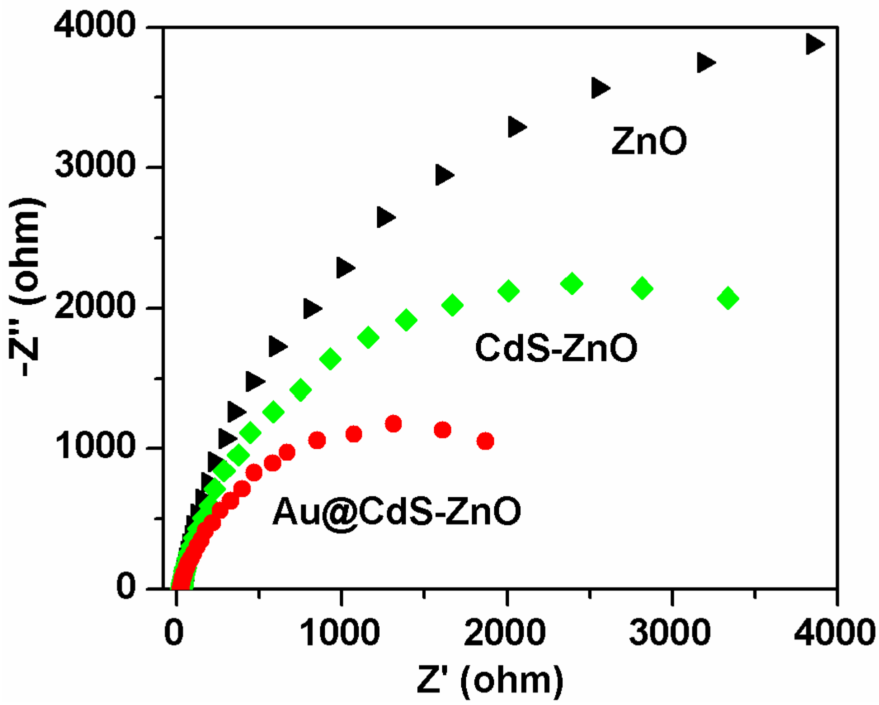

Figure S13. Electrochemical impedance spectroscopy (EIS) spectra of the PEC cells recorded at open circuit voltage under AM1.5 light irradiation.
